# Epidemiological models for the suicide rate of the Russian population in the period 1992-2022

**DOI:** 10.1192/j.eurpsy.2025.600

**Published:** 2025-08-26

**Authors:** A. Magay, V. Mitikhin

**Affiliations:** 1 Mental Health Research Centre, Moscow, Russian Federation

## Abstract

**Introduction:**

Suicides remain one of the most important problems of public health and society, being the most common reason for seeking emergency psychiatric care. The suicide rate is one of the most important indicators of the mental health of the population. Epidemiological models allow you to demonstrate the scale of problems and assess the resources to solve them.

**Objectives:**

The construction of epidemiological models for the suicide rate of the Russian population in order to analyze the relationship of this indicator with demographic and socio-economic factors in the period 1992-2022.

**Methods:**

The work uses data from Russian socio-economic statistics, materials from medical and research institutions and results published in scientific periodicals (see, [1] and references). In the formation of epidemiological models, the methods of systematic data analysis presented in the work [1] and statistical analysis in the framework of MS Excel were used.

**Results:**

During the period under review, for the Russian population, there is an almost linear dependence (negative) of the suicide rate on the main integral factor - life expectancy (years) (for the Russian population). The correlation coefficient of this factor with the suicide rate is 0.959. The next most important influence on the suicide rate is the unemployment rate (correlation coefficient 0.764). Using regression analysis, one-factor and two-factor epidemiological models for the suicide rate from these factors were obtained. The obtained regression models are characterized by very high reliability with a coefficient of determination R^2^ equal to 0.919 for the one-factor model and, accordingly, 0.943 for the two-factor model.

These results, obtained on the basis of data for the period of large-scale socio-economic and political reforms, allow us to offer a rational explanation for the observed rapid (in the period under review) deterioration of the mental health of the population. Conservative genetic mechanisms cannot fully explain such rapid variations in the mental health of the population. Consequently, the main weight in the study of mental disorders of the Russian population is given to demographic, socio-economic and cultural living conditions of the population, psychosocial stress, environmental factors and, possibly, epigenetic changes caused by them.

**Conclusions:**

The obtained models make it possible to quickly monitor and predict the impact of demographic, socio-economic factors and changes in the staffing of psychiatric care on the suicide rate of the Russian population. 1. Mitikhin V., Yastrebov V. et al. *Neuroscience and Behavioral Physiology.* 2019; 49(2): 233-239. doi: 10.1007/s11055-019-00720-4

**Disclosure of Interest:**

None Declared

